# Unlocking the potential of ‘passive’ modulation: How sensory stimulation shapes hand and face size

**DOI:** 10.1111/jnp.12379

**Published:** 2024-06-14

**Authors:** Laura Mora, Giorgia Committeri, Teresa L'Abbate, Gianna Cocchini

**Affiliations:** ^1^ Psychology Department Goldsmiths University of London London UK; ^2^ Institute of Advanced Biomedical Technologies University "G. d'Annunzio" Chieti‐Pescara Italy; ^3^ Department of Psychology International Telematic University Uninettuno Rome Italy

**Keywords:** body representation, body size distortions, body size modulation, multisensory integration, passive sensory stimulation, somatosensory information

## Abstract

Knowledge of the body size is intricately tied to multisensory integration processes that rely on the dynamic interplay of top‐down and bottom‐up mechanisms. Recent years have seen the development of passive sensory stimulation protocols aimed at investigating the modulation of various cognitive functions, primarily inducing perceptual learning and behaviour change without the need for extensive training. Given that reductions in sensory input have been associated with alterations in body size perception, it is reasonable to hypothesize that increasing sensory information through passive sensory stimulation could similarly influence the perception of the size of body parts. The primary aim of this study was to investigate the potential modulatory effects of passive sensory stimulation on the perception of hand and face size in a group of young adults. Passive sensory stimulation effectively modulated the size representation of the stimulated hand, supporting the notion that access to somatosensory and proprioceptive information is prioritised for the hands but may not extend to the face. Increased somatosensory input resulted in a reduction of distortion, providing evidence for bottom‐up modulation of size representation. Passive sensory stimulation can induce subjective changes in body size perception without the need for extensive training. This paradigm holds promise as a potential alternative for modulating distorted size representation in individuals with body representational deficits.

## INTRODUCTION

It is well known that implicit knowledge of the size of our body is essential for movement and interactions with the environment (Gandevia & Phegan, 1999). For instance, the length of the arms needs to be computed in order to reach an object (Coelho & Gonzalez, 2018a; Longo & Lourenco, 2007), the length of our legs to walk (Stone et al., 2018), and the height and width of our body to go through a door (Stefanucci & Geuss, 2010). The importance of holding an accurate size representation of our body is perhaps better understood when considering the impact that size distortions can have on daily functioning. For example, telescoping, an altered perception of limb size, has been linked to increased pain in amputees experiencing phantom limbs (Schmalzl & Ehrsson, 2011). Similarly, distorted information about the size of our body is linked to a variety of pathologies, such as anorexia nervosa (AN), in which patients experience an oversized body (Gadsby, 2017; Riva, 2012; Slade & Russell, 1973).

Body size representation crucially depends on multisensory integration (Azañón et al., 2016; De Vignemont, 2014), relying on top‐down and bottom‐up mechanisms that are constantly in interaction to build a coherent representation (Di Russo et al., 2006; Di Vita et al., 2016; Longo, 2015; Palermo et al., 2014; Pitron et al., 2018; Serino & Haggard, 2010). Indeed, it is generally agreed that in order to build body awareness, bottom‐up information needs to be collected from multiple sources, such as touch, proprioception, vestibular system and vision (De Vignemont, 2010). This information is then integrated with top‐down processes and utilised to conform to the different aspects of body representation (Buxbaum & Coslett, 2001; Sirigu et al., 1991). Interestingly, this representation is malleable (Stone et al., 2018) when manipulating these processes. For instance, afferent sensory information affects the size of a perceived body part almost instantly after acute decreases (anaesthesia) or increases (electrical cutaneous stimulation) of sensory input (Gandevia & Phegan, 1999). Bottom‐up modulation through anaesthesia increases the perceived size of body parts, such as the thumb (Gandevia & Phegan, 1999; Paqueron et al., 2003), the lips and teeth (Türker et al., 2005) or the upper and lower limbs (Paqueron et al., 2003). This is due to increased anomalous efferent discharge after removal of afferent information, which is associated with reduced cortical activity in the somatosensory area (Gandevia & Phegan, 1999). Similarly, top‐down modulation through visual magnification or minification of the size of body parts affects reach and grasp (Ambron et al., 2017; Marino et al., 2010) or tactile perception (Taylor‐Clarke et al., 2004). Passive sensory stimulation protocols have been developed in recent years to study the modulation of different cognitive functions, mainly to induce perceptual learning and behaviour change without training (Beste & Dinse, 2013; Dinse et al., 2003). This approach has proven useful to improve sensorimotor functions in health (Ladda et al., 2014) or mobility in old age (Kalisch et al., 2008, 2010). It is based on the use‐dependent plasticity knowledge and multisensory integration principles (Baumard & Osiurak, 2019). The modulation of perceptual functions is achieved by implementing the so‐called coactivation protocol, in which repetitive synchronous stimulation is administered by a solenoid that simultaneously activates a large number of receptive fields. This stimulation increases neural activity boosting the somatosensory representation and eliciting plastic changes, mimicking what occurs after training or learning (Beste & Dinse, 2013; Pleger et al., 2001). This, in turn, improves sensorimotor functions, such as tactile spatial discrimination (Dinse et al., 2003, 2011; Kalisch et al., 2008; Ladda et al., 2014; Pleger et al., 2001, 2003), motor function after stroke (Wu et al., 2006) or mobility in old age (Kalisch et al., 2008, 2010). Specifically, the level of amelioration is strongly correlated with the degree of cortical reorganisation in the primary somatosensory cortex; that is, larger reorganisation commensurate to greater behavioural gains (Kalisch et al., 2007; Pleger et al., 2001, 2003).

The action mechanism of passive sensory stimulation is associated with increased cortical excitability after a short period of somatosensory stimulation (Kaelin‐Lang et al., 2002; Ridding et al., 2000), with intracortical facilitation (Kobayashi et al., 2003) and decrease of inhibition (Classen et al., 2000). In particular, excitability changes are associated to GABAergic neurotransmission (Kaelin‐Lang et al., 2002) and LTP mechanisms (Stefan, 2000). Inversely, deafferentation causes changes in cortical motor excitability (Ziemann, 2001), associated with a reduction of GABA levels (Levy et al., 2002). Moreover, the effects of vibration are not circumscribed to somatosensory areas. In fact, vibratory stimulation applied to the hand (palm) increases regional cerebral blood flow (rCBF) in contralateral primary and secondary somatosensory areas, parietal cortex and primary and supplementary motor areas (Seitz & Roland, 1992). Hence, it appears to engage several areas that are also involved in the representation of the body size.

Reduced sensory input (due to lesions or anaesthesia) has been associated with perceived body size modulation (Gandevia & Phegan, 1999; Paqueron et al., 2003; Türker et al., 2005), suggesting a central role of sensory information on body representation. Therefore, we expected that some degree of subjective distortion of body size can result also by increasing sensory information (e.g. by means of passive sensory stimulation). Herein, our aim was to study the potential modulatory effect of passive sensory stimulation on body size perception of hands and face. Specifically, our goal was to discern how sensory modalities (i.e. somatosensation) predominantly influence different body parts, and not others, to construct a unified representation. Previous research has shown that the size representation of a body part is primed by the sensory modality that is most prominently used to perceive it (Stone et al., 2018). For instance, visual experience of a body part that relies less on somatosensation (i.e. legs), appears more relevant in the representation of their size with characteristic distortions. Instead, these are not reported in other body parts (i.e. hands) in which somatosensory (tactile) information is of more relevance to represent them (Stone et al., 2018; Weinstein, 1968). As tactile acuity varies across faces and hands (Mancini et al., 2014; Weber & Ross, 1978; Weinstein, 1968), it is possible that vibration will have different effects across these two body parts.

## METHOD

### Participants

An a priori power analysis was run to determine the required sample size by using G* Power 3.1 (Faul et al., 2009). A power analysis based on an ANCOVA with two covariates was run. We considered an average effect size f of .8, based on previously published studies (e.g. Caggiano & Cocchini, 2020; Mora et al., 2018). Alpha was set at .05 and a power of .95. The adequate sample size obtained was 23.

A group of 30 healthy volunteers (20 females and 10 males) was recruited to take part in this study. Half of them (*n* = 15, 10 females and 5 males in each group) were randomly assigned to the face stimulation group, while the other half were part of the hand stimulation group (see Demographic information in Table 1). Groups did not differ in age [*t* (28) = .33, *p* = .74, *d* = .12] or formal education [*t* (28) = −.59, *p* = .56, *d* = −.22]. There were two left‐handed participants in each group, as measured with the Oldfield questionnaire (Oldfield, 1971), and handedness did not differ between groups either [*t* (28) = −.17, *p* = .86, *d* = −.06].

**TABLE 1:** Demographic table.

	Face stimulation group (*N* = 15)	Hand stimulation group (*N* = 15)
Age (years)		
Mean	24.4	24.13
SD	2.53	1.85
Range	22–31	22–28
Education (years)		
Mean	16.67	17
SD	1.63	1.41
Range	13–18	15–20
Edinburgh Handedness Inventory		
Mean	.61	.65
SD	.55	.59
Range	−.89–1	−.89–1
Body Shape Questionnaire (BSQ)		
Mean	64.6	75.13
SD	23.37	24.32
Range	36–103	49–123
Vividness of Visual Images Questionnaire (VVIQ)		
Mean	57.6	59.2
SD	7.46	8.83
Range	46–74	45–76

### Related measures

Previous research has found differences in body size estimation depending on mental imagery (Auchus et al., 1993), the skill needed to judge the size of one's own body (Auchus et al., 1993; Darling et al., 2015; Smeets et al., 2009). Moreover, size‐distorted pictures, such as the ones used in this study, activate the mental representation of the body (Mohr et al., 2010; Spitoni et al., 2013). Further, body dissatisfaction, or ‘feeling fat’, has been shown to influence body size estimation (D'Amour & Harris, 2019; Mohr et al., 2011, 2016; Salvato et al., 2019; Smeets et al., 2009). This effect also appears in the size estimation of otherwise presumed ‘immune’ body parts such as the hands (Coelho & Gonzalez, 2018b). To control for possible between‐group differences and to consider the influence on body size representation, these variables were measured. Specifically, participant's body dissatisfaction was measured by means of the Body Shape Questionnaire (BSQ) (Cooper et al., 1987), while their visual imagery skills were evaluated using the Vividness of Visual Images Questionnaire (VVIQ) (Marks, 1973).

The BSQ includes 34 items focusing on the experience of ‘feeling fat’ for the previous 4 weeks, and each was evaluated with a 6‐point Likert scale (from ‘never’ to ‘always’). Thus, scores ranged from 34 points (no concern) to 204 (high concern). Higher scores are indicators of higher body dissatisfaction.

The VVIQ consists of 16 items to visualise, such as the face of a friend or relative. The vividness of the mental image produced is then rated on a Likert‐type scale (1 meaning no image at all and 5 meaning a perfectly clear and vivid as real image), with a maximum score of 80, and a minimum of 16. Low scores will indicate poor imagery, while high scores are indicative of stronger visual imagery skills.

### Passive sensory stimulation protocol

Interestingly, attentional factors, rather than sensory information, have been found key for body size representation in a recent study in which sensory stimulation was administered (Caggiano et al., 2023). However, this result could have been due to the lack of sensitivity of their sensory stimulation paradigm as single brief vibrations were applied of short length (~1 s) and somatosensory information may need longer exposure to induce a significant change in the perceived body size. Hence, a longer duration of passive sensory stimulation (20 min) that co‐activates a larger number of receptors could be a better candidate to explore this limitation.

**FIGURE 1:**
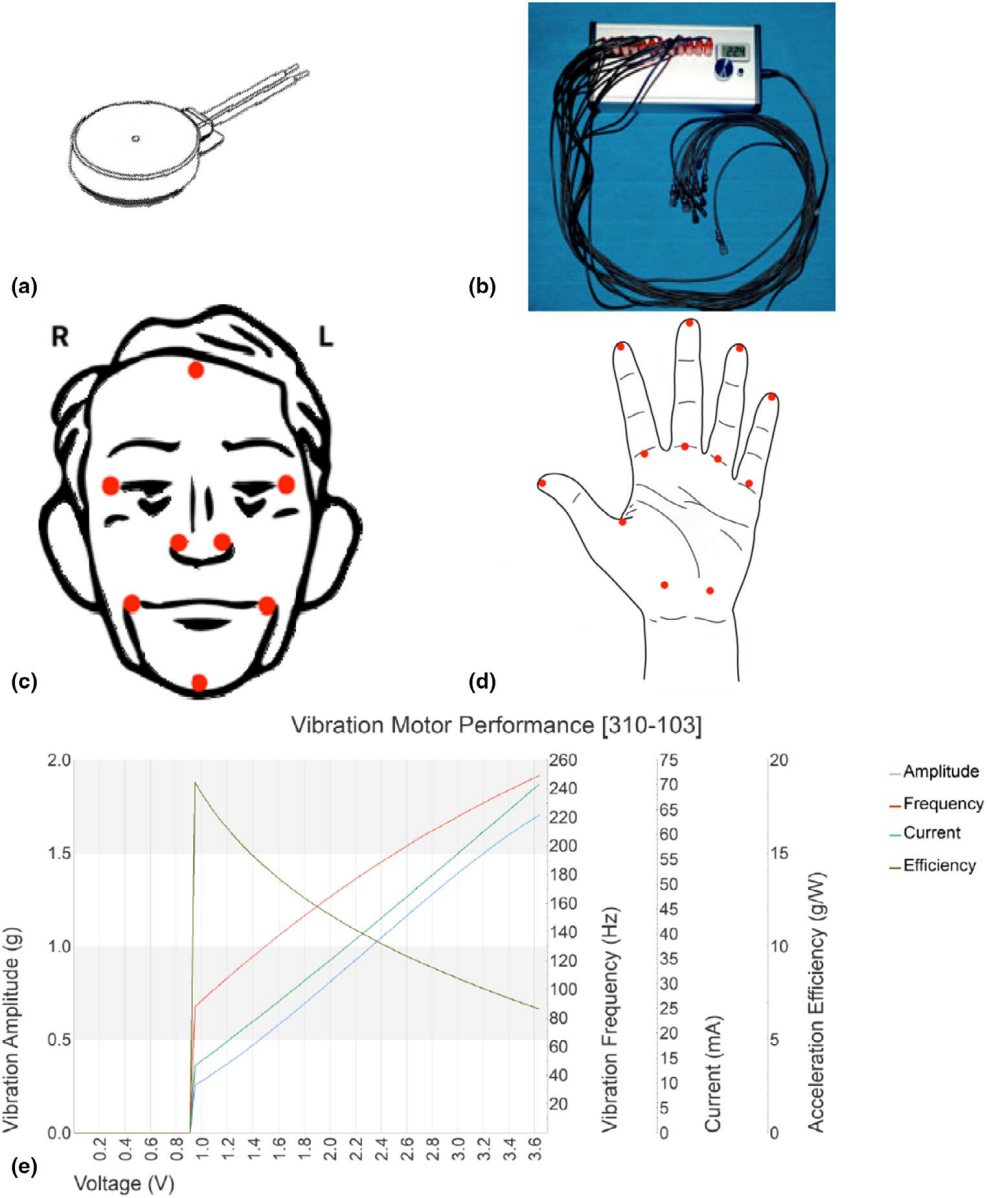
Passive sensory stimulation device and procedure. Drawing of vibration motor model 310‐103 (a), picture of experimental portable vibration device (b), location of eight vibration motors for the face (c) and 12 vibration motors for the hand (d); and typical performance characteristics of the vibration motors, showing vibration amplitude (g), voltage (V), vibration frequency (Hz), current (mA) and acceleration efficiency (g/W) (e). a and e reproduced from Precision Microdrives product data sheet with permission.

A bespoke‐made experimental portable device was used to deliver the passive sensory stimulation through circular vibration motors (Figure 1a) from Precision Microdrives Ltd (type 310‐103). These were powered by an adjustable voltage supply built in‐house (see picture of equipment in Figure 1b). It is crucial that stimulation targets/coactivates a large number of receptors (solenoid of around 8 mm), as single‐site stimulation (.8 mm^2^ solenoid) has not been found to elicit any activation or perceptual changes (Pleger et al., 2003). Thus, in this study, 10‐mm‐sized vibration motors were used. Further, different areas were stimulated as single‐finger stimulation has been shown not to extend effects to adjacent or contralateral fingers (Gandevia & Phegan, 1999). Synchronous stimulation was provided, as this is instrumental for multisite stimulation (Kalisch et al., 2007). A total of eight motors were used for the face stimulation and 12 for the hand (see location of vibration motors in Figure 1c,d) that were attached with medical tape. The choice of eight motors for face stimulation and 12 motors for hand stimulation was driven by the need to balance coverage of relevant landmarks without compromising participant's comfort. Fewer motors were used for the face to avoid discomfort, while a greater number were used for the hand to ensure comprehensive stimulation of its various sensory regions. The hand stimulation was administered in the dominant hand. The current (mA) was adjusted to control the frequency of the stimulation (see graph with motor performance characteristics in Figure 1e). High‐frequency stimulation (over 10 Hz) was delivered as this has been shown to elicit long‐term potentiation (LTP) of brain activity, associated with perceptual gains (Beste & Dinse, 2013). Frequencies higher than 50 Hz are considered vibration (Francis et al., 2000). The intensity of vibration was set individually at the highest level the participant could comfortably tolerate for a duration of 20 min, given that larger improvements in perceptual abilities seem to follow higher intensity of sensory stimulation (Beste & Dinse, 2013; Ladda et al., 2014; Schlieper & Dinse, 2012).

Participants were told the stimulation could be stopped at any time if they felt any discomfort. All participants were able to tolerate 20 min of stimulation. Some habituation to the stimulation was observed halfway through the procedure as participants reported to feel a reduction in the intensity. After stimulation, participants experienced ‘vibration aftereffects’ for a few minutes, as reported in previous studies (Ladda et al., 2014); only one participant reported some discomfort after stimulation (numbness of the face). Perceptual illusions, such as the Pinocchio illusion, which entail perceived limb movement or conscious changes in shape and size, have been successfully induced through vibratory stimulation at the level of tendons of joint extensor muscles (De Vignemont et al., 2005; Lackner, 1988). Notably, participants in our study did not report experiencing illusory movements either during or after stimulation. Consequently, we consider it improbable that the results of this study could be attributed to such perceptual phenomena.

### Body size estimation task

The size estimation task has been inspired by the tasks used in previous research (Gandevia & Phegan, 1999; Longo & Haggard, 2012; Mora et al., 2023; Türker et al., 2005) and consists of presenting distorted pictures of body parts for the participants to assess which one would match their perceived body size (Gardner & Boice, 2004). This task has been reliably used in previous experiments manipulating size perception in health (Gandevia & Phegan, 1999; Paqueron et al., 2003) and in illness, such as in anorexia nervosa (Mohr et al., 2016) or recently in personal neglect (Mora et al., 2023). Similar tasks have shown that healthy participants show distortions in size perception with wider and shorter faces (D'Amour & Harris, 2017), smaller hands and feet (Giurgola et al., 2019), overestimated eyes (Hine & Okubo, 2021) or even a stouter body in professional swimmers (Urdapilleta et al., 2010). This task was considered most appropriate as administering passive sensory stimulation could interact with other size estimation tasks, such as proprioceptive pointing.

In this study, single body parts were presented to avoid comparative judgements (Fuentes et al., 2013). Moreover, real‐sized pictures of participants' own bodies were presented as results are susceptible to less distortion due to procedural confounds (Cullari et al., 2002; Holder & Keates, 2006).

#### Stimuli

One photograph of the face (with neutral expression) and one of the palms of the right hand with fingers spread out were taken of each participant at a 1 m distance by using a Fujifilm Finepix HS 25EXR camera. The focal point of the camera was focused on the centre of the body part (tip of the nose for the face, centre of the palm for the hand). The background from the pictures was removed and set as standard white by using Windows Paint 3D programme, to prevent providing any cues (Gardner & Boice, 2004). Any connection to the body (i.e., the neck or arm) was also removed, leaving a picture of just the head or the hand. The face picture was reversed to act as a mirrored image of the participant's face, as this is how they would normally see it (as in D'Amour & Harris, 2017). The image of the right hand was mirrored to produce an image of the left (as per previous studies).

**FIGURE 2:**
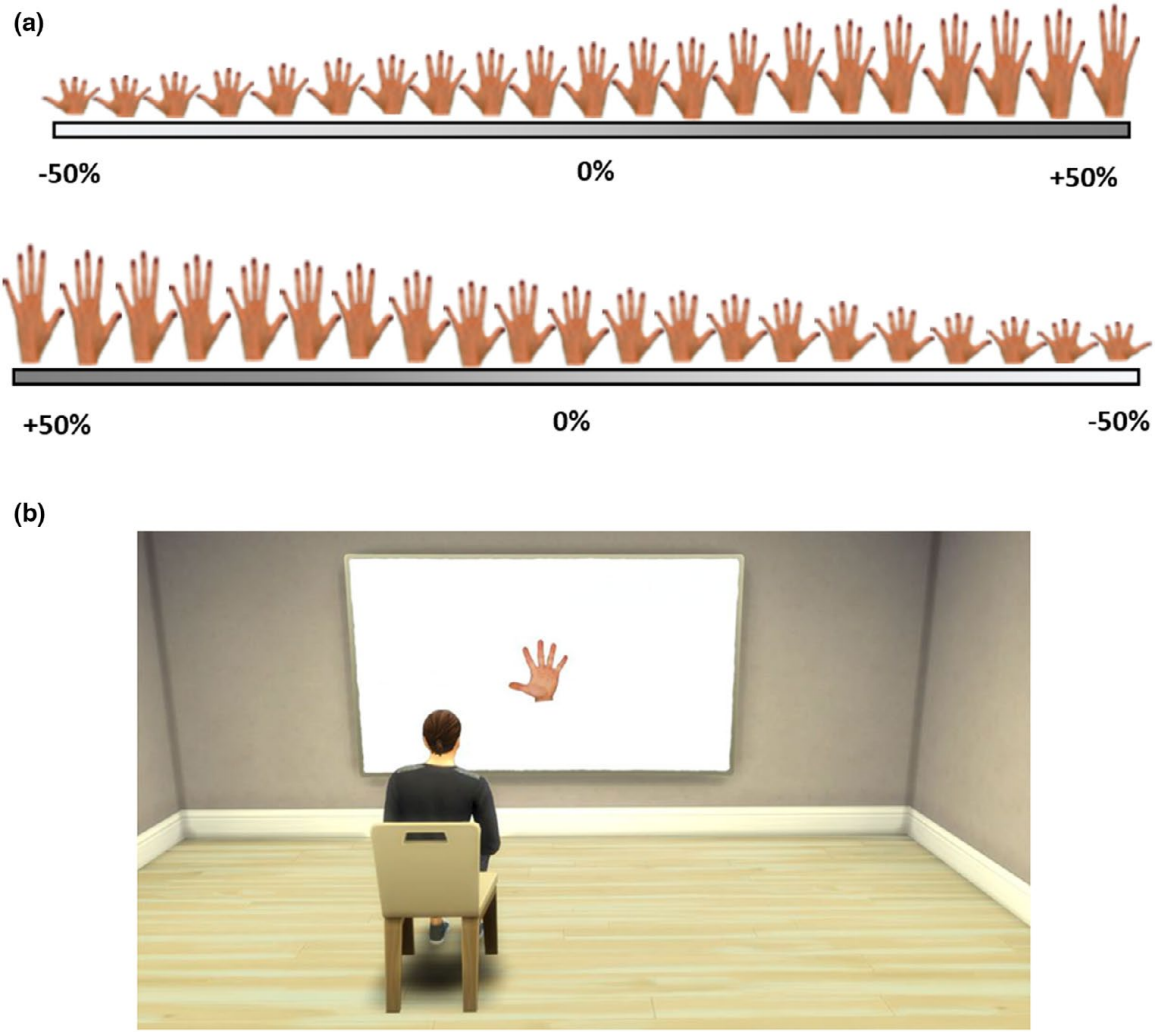
Experimental setting. Right‐hand pictures in ascending order (small to big) and left‐hand pictures in descending order (big to small) with 5% size changes intervals for length (a), pictorial representation of the experimental setting (b).

The final images were then distorted in different dimensions (width and length), as in previous studies (Gardner et al., 1995; Gardner & Brown, 2014; Mohr et al., 2016; Van der Looven et al., 2021). The intention was to prevent participants from potentially relying solely on the body part's position relative to their view to perceive correct proportions (i.e. seeing the body part closer or farther from them). For this, a bespoke‐designed programme created with Borland C^++^ builder (2007) was used. Distortions were introduced in 5% intervals, with symmetrical distortion from the midline of each body part. The smallest‐sized picture was 50% smaller than the real‐sized picture, while the largest picture was 50% larger (thus, from 50% to 150% distortion). There was a total of 21 pictures for the face, and 21 pictures for each hand (a total of 63 pictures for all conditions in each dimension), with only one being the real‐sized picture for each body part (100% size). See a hand presentation example in Figure 2a.

#### Experimental procedure

Participants sat in a chair about 1 m from a wall. The pictures were projected onto a white screen in real size by using a projector, model NEC NP07LP, connected to a laptop used to present the pictures. The projector was positioned at approximately 1.8 m to the wall (behind the participant), on a table at 1 m of height (see Figure 2b). The projected image was adjusted in a way that the real‐sized picture (100% sized, i.e. no distortion) matched the real size of the participant's face or hands. For this, the size of the body parts was measured with a measuring tape and matched to the projected undistorted image onto the wall. The images were projected on the right visual hemispace to avoid obstruction of the stimuli projection caused by the participant's head.

One picture was presented at a time. There were two presentation orders: ascending (i.e. from smallest to largest picture) and descending (from largest to smallest). Half of the participants started with an ascending trial, and the other half with a descending one. The ascending and descending orders were alternated to control for order effects (Auchus et al., 1993). Pictures were presented twice in each presentation order (ascending and descending) and for two dimensions (length and width), counterbalanced (a total of 8 trials per body part). Participants were asked to decide (‘yes/no’) if the picture presented on the wall matched their real sized body part, according to each dimension. If their response was negative, the experimenter presented the next picture of the sequence. The procedure stopped when participants confirmed that the picture projected corresponded to be their veridical size. Participants performed this task twice in this experiment: before and after the passive sensory stimulation, and for both body parts (i.e., hands and face). Overall, each participant responded to 24 sequences (before and after stimulation). During the testing session, participants' hands were kept out of view by a screen.

### Dexterity assessment

Repetitive sensory stimulation can improve motor performance, in particular when used for longer stimulation periods (Kalisch et al., 2008; Ladda et al., 2014). In order to study the potential effect of passive stimulation on motor performance, the Nine‐hole peg test was administered (Kellor et al., 1971; Mathiowetz et al., 1985). The cardboard version produced by clinicspeak.com was used (Dubuisson et al., 2017). This version provides a measure of finger dexterity by counting the time it takes for the participant to put the nine wooden pegs (6 × 30 mm) into nine holes, one at a time, and then remove them. The procedure is performed with both the dominant and the non‐dominant hands (Mathiowetz et al., 1985). The test was administered before and after passive stimulation.

### General analyses

The *Body Size Distortion* was calculated considering the order of presentation by obtaining the percentage of over/underestimation for each body part. Previous studies have identified an asymmetry in the perception of size depending on the presentation order due to cognitive biases (Auchus et al., 1993; Mora et al., 2023) and showed that averaged size judgements do not accurately represent the real performance (Gardner & Boice, 2004; Gardner & Bokenkamp, 1996). These studies reported that overall performance is more accurate in ascending presentations (Gardner & Bokenkamp, 1996). Thus, the size estimation results were analysed separating performance in ascending and descending conditions, averaged across body parts.

### Statistical analyses

To start, we employed two‐sample t‐tests to compute disparities in the related measures (specifically, body dissatisfaction and mental imagery) as well as the intensity of passive sensory stimulation between the groups. Subsequently, we conducted mixed‐model ANCOVAs to examine the *Body Size Distortion*, incorporating VVIQ and BSQ scores as covariates to account for possible confounding effects. The data from each presentation order (both ascending and descending) were separately analysed to gain insight into the direction of the distortion, in accordance with the approach outlined by Gardner and Boice (2004), Gardner and Bokenkamp (1996) and Mora et al. (2023). Similarly, we analysed each body part separately to discern the distinct effects of stimulation. Thus, there were two key factors under investigation: Time (assessed at both pre‐ and post‐stimulation time points) and Group (face or hand stimulation). In instances where significant interactions were detected in the ANCOVA, we conducted Bonferroni‐corrected t‐tests to further explore these effects.

Lastly, dexterity assessment results were then presented and analysed via a mixed‐model ANOVA with three factors: time (pre‐ and post‐), hand (dominant and non‐dominant) and group (face or hand stimulation).

## RESULTS

### Related measures effects

We calculated overall differences in body dissatisfaction (BSQ) and mental imagery (VVIQ) between groups. Differences between groups were not significant in BSQ [*t* (28) = −1.21, *p* = .24, *d* = −.44] or VVIQ [*t* (28) = −.54, *p* = .59, *d* = −.2], confirming their performance was equivalent.

### Passive sensory stimulation

Not surprisingly, the intensity of vibration that participants were able to tolerate differed between stimulation groups (i.e. stimulated body part). Specifically, the average intensity of stimulation for the face stimulation group that participants were able to tolerate was of 17.49 mA (SD = 3.84), which corresponds to an average frequency of 60 Hz. In contrast, the current intensity for the hand group was of 33.02 mA (SD = 3.42) and an average frequency of 110 Hz (see graph in Figure 1e). Differences in the intensity of stimulation were significant between groups [*t* (28) = −11.71, *p* < .001, *d* = −4.28].

### Body size estimation task

Mean perceived size distortion (percentage of under/overestimation) for all body parts confirmed that there was underestimation in the ascending presentations and overestimation in the descending ones. This fact justified the need to separate the analyses for ascending and descending conditions (Gardner & Boice, 2004; Gardner & Bokenkamp, 1996).

#### Face size distortion

**FIGURE 3:**
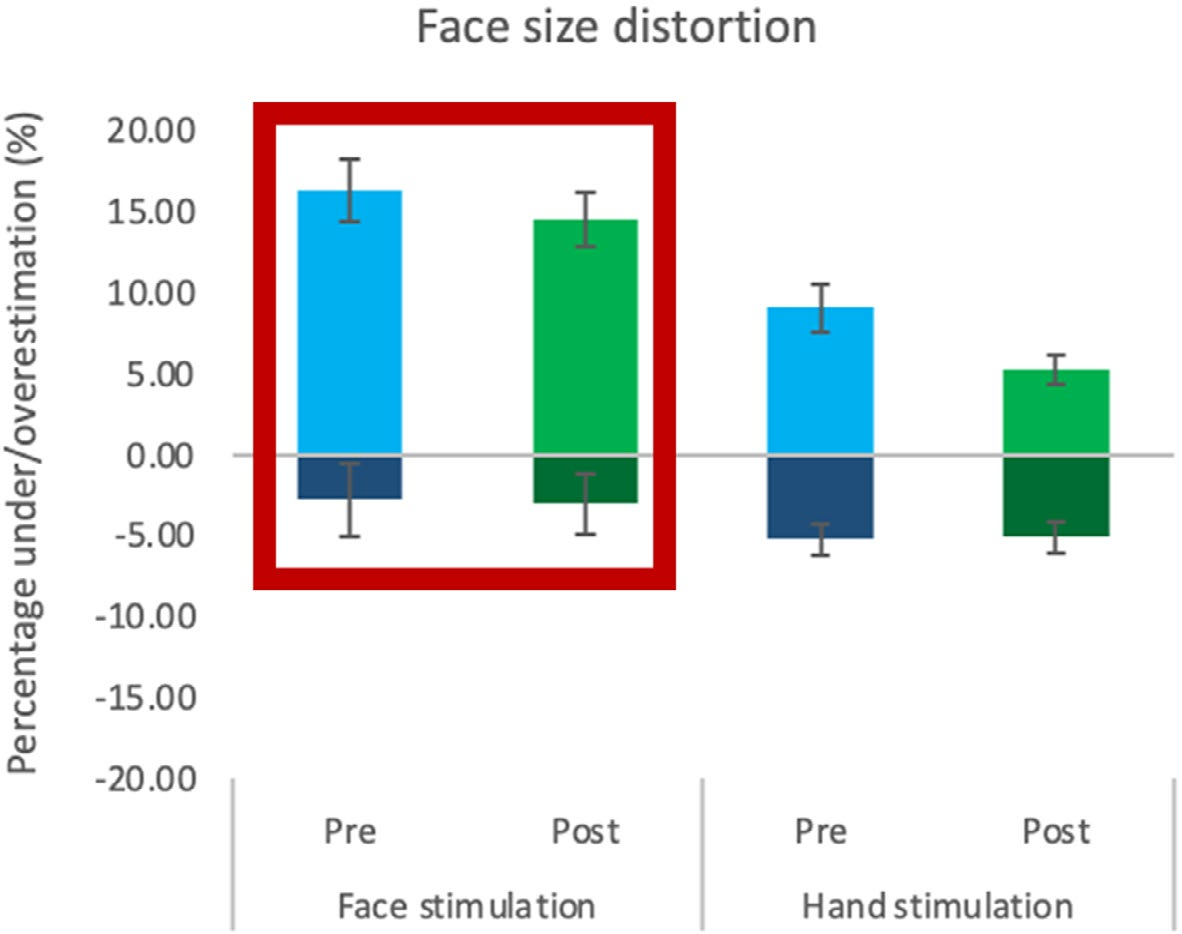
Face size distortion before and after stimulation. Representation of the perceived distortion (percentage of over/underestimation) for the size of the face before and after stimulation for face stimulation and hand stimulation groups. The presentation order is indicated by darker colour bars at the bottom (ascending) and lighter colour bars on top (descending). The red rectangle highlights which group received stimulation on the face. Error bars indicate the standard error of the mean.

**FIGURE 4:**
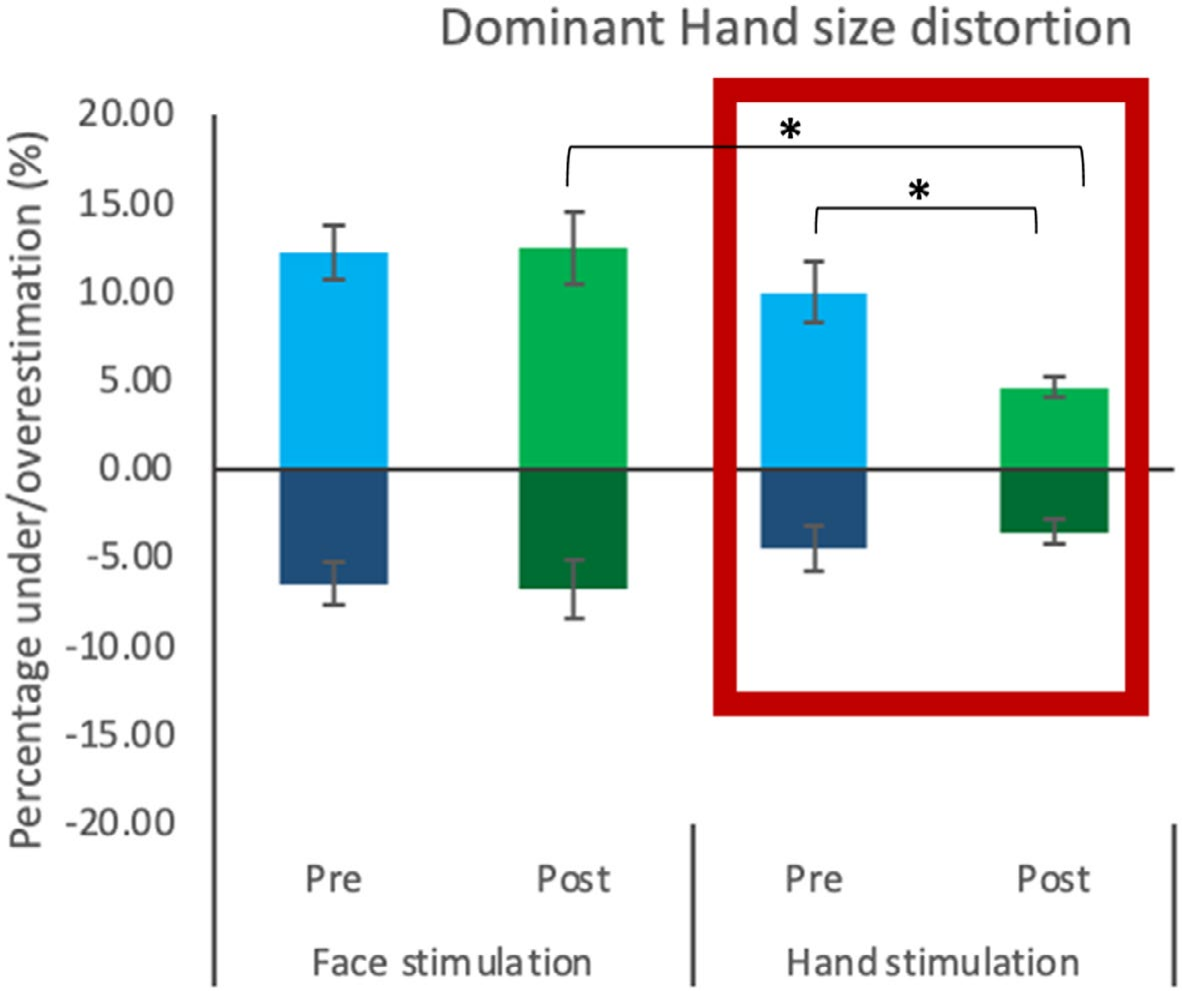
Dominant hand size distortion before and after stimulation. Representation of the perceived distortion (percentage of over/underestimation) of the dominant hand before and after stimulation, for face stimulation and hand stimulation groups. The presentation order is indicated by darker colour bars at the bottom (ascending) and lighter colour bars on top (descending). The red rectangle highlights which group received stimulation on the dominant hand. Error bars indicate the standard error of the mean. *Significant differences (*p* < .01).

The mixed‐model ANCOVA for the ascending presentation detected a non‐significant effect of Time [*F* (1, 26) = .73, *p* = .4, $\eta_{p}^{2}$ = .03], Group [*F* (1, 26) = .58, *p* = .45, $\eta_{p}^{2}$ = .02] and Time by Group interaction [*F* (1, 26) = .32, *p* = .58, $\eta_{p}^{2}$ = .01]. In the descending order presentation (lighter colour bars in Figure 4), the Time factor was not significant [*F* (1, 26) = 1.06, *p* = .31, $\eta_{p}^{2}$ = .04], whereas the factor Group was [*F* (1, 26) = 15.33, *p* = .001, $\eta_{p}^{2}$ = .37]. Overall, the face stimulation group showed more distortion (*M* = 15.46%, SD = 6.71) than the hand group (*M* = 7.17%, SD = 4.22). Lastly, the interaction between Time and Group was not significant [*F* (1, 26) = 2.33, *p* = .14, $\eta_{p}^{2}$ = .08]. Taken together, these results indicated that there was no effect of stimulation in the representation of the face (see Figure 3).

#### Dominant hand size distortion

In the ascending condition, Time [*F* (1, 26) = .24, *p* = .63, $\eta_{p}^{2}$ = .01], Group [*F* (1, 26) = 84.46, *p* = .14, $\eta_{p}^{2}$ = .08] and Time by Group interaction [*F* (1, 26) = .98, *p* = .33, $\eta_{p}^{2}$ = .04] were not significant.

For the descending order, the effect of Time was not significant [*F* (1, 26) = .24, *p* = .63, $\eta_{p}^{2}$ = .01], whereas Group [*F* (1, 26) = 8.15, *p* = .01, $\eta_{p}^{2}$ = .24] and Time by Group interaction [*F* (1, 26) = 5.37, *p* = .03, $\eta_{p}^{2}$ = .17] were. The significance of the Group factor confirmed differences in size perception between groups, being the hand stimulation group the most accurate, overall (see Figure 4). To explore the interaction between Time and Group, four Bonferroni‐corrected t‐tests were run (a cut‐off *p*‐value of <.01); two between the size perception before and after stimulation within each group (pairwise comparisons), and two between size perception pre‐ and post‐stimulation between groups (independent group comparisons). Pre‐ and post‐stimulation differences were minimal and not significant for the face stimulation group [*t* (14) = −.13, *p* = .9, *d* = −.03]. In contrast, differences in the hand group reached significance [*t* (14) = 3.6, *p* = .003, *d* = .93]. Differences between groups pre‐stimulation were not significant [*t* (28) = .99, *p* = .33, *d* = .36], whereas these were significant post‐stimulation [*t* (16.41) = 3.65, *p* = .002, *d* = 1.33] (equality of variances not assumed). In this case, these results indicated that there was a significant effect of stimulation in the representation of the dominant hand, with a reduction of the baseline distortion.

#### Non‐dominant hand

As seen in previous body parts, in the ascending presentation results did not reach significance for any factor (Time: [*F* (1, 26) = .003, *p* = .96, $\eta_{p}^{2}$ = .00]; Group: [*F* (1, 26) = .001, *p* = .97, $\eta_{p}^{2}$ = .00]), indicating no differences between groups or measurements. Equally, the interaction between Time and Group was not significant [*F* (1, 26) = 3.75, *p* = .06, $\eta_{p}^{2}$ = .07]. Thus, there were no effects of stimulation in the ascending condition.

**FIGURE 5:**
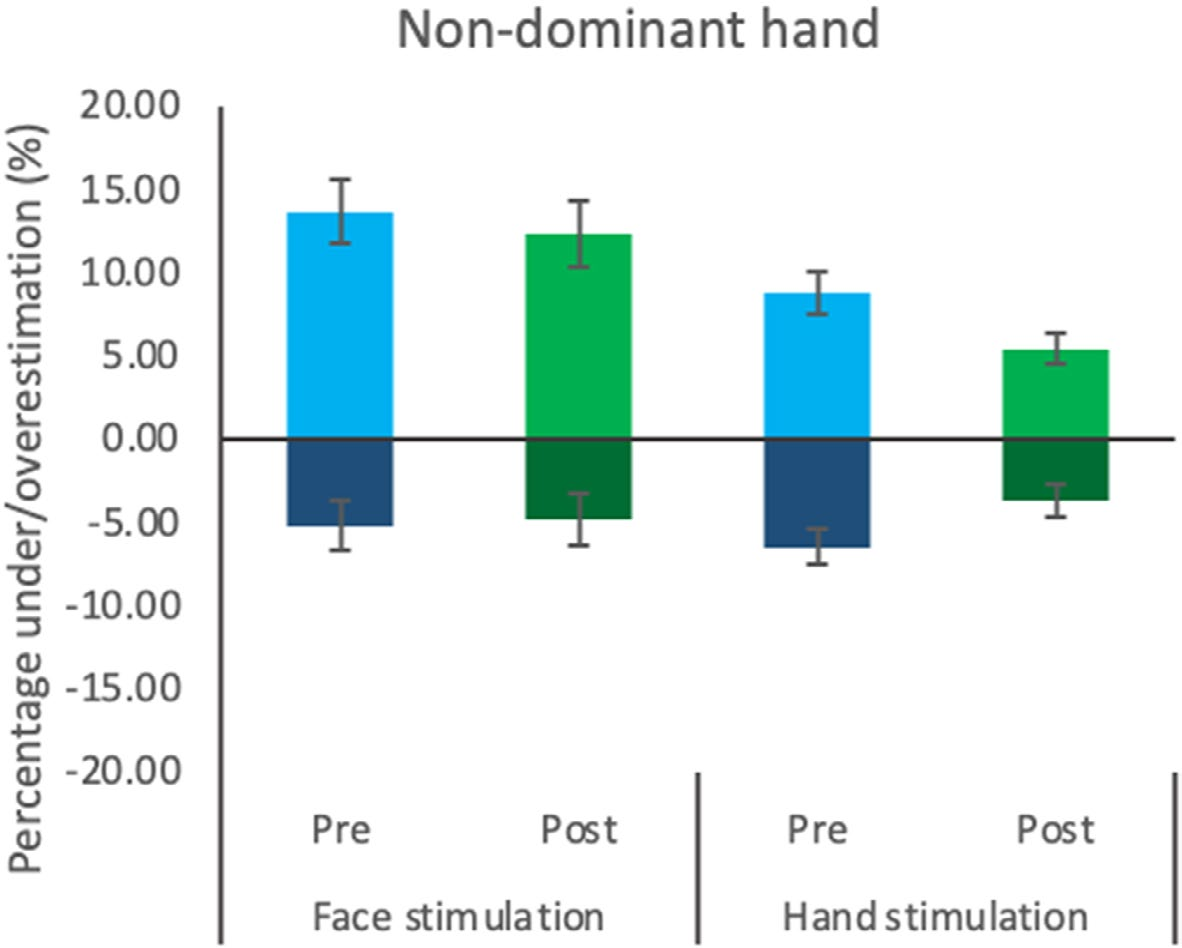
Non‐dominant hand size distortion before and after stimulation. Representation of the perceived distortion (percentage of over/underestimation) of the non‐dominant hand before and after stimulation, for the face stimulation and hand stimulation groups. The presentation order is indicated by darker colour bars at the bottom (ascending) and lighter colour bars on top (descending). Error bars indicate the standard error of the mean.

Results for the descending presentation did not show significant effects for the factor Time [*F* (1, 26) = 3.56, *p* = .07, $\eta_{p}^{2}$ = .12] nor for the Time by Group interaction [*F* (1, 26) = 2.54, *p* = .12, $\eta_{p}^{2}$ = .09], but the Group factor was significant [*F* (1, 26) = 7.35, *p* = .01, $\eta_{p}^{2}$ = .22], confirming that the face stimulation group was less accurate, overall (see Figure 5). However, the lack of interaction effect does not support a difference in size distortion due to stimulation effects. To sum up, these results confirmed the lack of effect of the stimulation on the non‐dominant hand.

#### Dexterity

To test the effect of passive stimulation on dexterity, the pre‐ and post‐stimulation results on the Nine‐hole peg test were considered for each group. An improvement in performance was predicted after stimulating the dominant hand, following previous studies reporting improved motor performance after hand stimulation (e.g., Ladda et al., 2014). No effect on dexterity was anticipated after face stimulation. Results are presented in Table 2.

**TABLE 2:** Nine‐hole peg test results. Performance in the dexterity test (in seconds) with standard deviation (SD), pre‐ and post‐stimulation for both hands and groups.

	Time	Face stimulation group	Hand stimulation group
		Mean (SD)	Mean (SD)
Dominant hand	Pre	18.06 (2.64)	19.82 (1.74)
	Post	17.49 (2.01)	18.94 (2.04)
Non‐dominant hand	Pre	18.57 (2.39)	20.41 (2.45)
	Post	18.08 (2.08)	20.51 (2.49)

The ANOVA did not show significant effects of Time [*F* (1, 28) = 2.93, *p* = .1, $\eta_{p}^{2}$ = .1]; whereas the main factor Hand was significant [*F* (1, 28) = 6.97, *p* = .01, $\eta_{p}^{2}$ = .2]. There was an overall better performance when using the dominant hand in comparison with the non‐dominant (mean difference = .81). Further, there were significant Group differences [*F* (1, 28) = 8.07, *p* = .008, $\eta_{p}^{2}$ = .22], with faster execution in the Face stimulation group (mean difference = 1.87). The interactions between Time and Group factors (*p* = .8); Hand and Group (*p* = .39); Time and Hand (*p* = .33); and Time, Hand and Group (*p* = .4), were all not significant. These results did not identify any effect of stimulation on dexterity performance, measured with the Nine‐hole peg test.

## DISCUSSION

In this study, the modulatory effects of passive sensory stimulation in the representation of the size of the body image of hands and face have been explored in healthy young volunteers by using an experimental bespoke‐made device. By applying passive sensory stimulation to either the face or hands, we aimed to modulate the size representation of these body parts. In the face stimulation group, we did not find an effect of stimulation on size perception. On the contrary, we found a modulation of the body size in the dominant hand stimulation group, with overall reduction of the distortion for the stimulated hand.

At the outset, participants were not accurate when estimating the size of hands and faces from pictures, with distortions both in ascending and descending presentations. Indeed, distortions of body image are found in healthy populations, with a tendency to overestimate the body (D'Amour & Harris, 2017; Urdapilleta et al., 2010). As expected, the presentation order (ascending vs. descending) had an influence on size judgements, which were influenced by the preceding one (Caggiano & Cocchini, 2020; Gardner & Boice, 2004; Gardner & Bokenkamp, 1996). In essence, the participants displayed lower accuracy during descending trials, which confirms the presence of preferential aftereffects following exposure to enlarged images. These aftereffects seemed to influence participants to select more distorted (enlarged) pictures compared to their choices in ascending trials (Gardner & Bokenkamp, 1996). This preference, also seen in embodiment (Haggard & Jundi, 2009; Pavani & Zampini, 2007), may be attributed to representational flexibility to accommodate growth through development (De Vignemont et al., 2005). Considering these findings at baseline, it is not surprising to find that the effects of stimulation are only evident in the descending order. Indeed, the ascending condition could be ‘at ceiling’.

Interestingly, passive sensory stimulation was effective at modulating the size representation of the body, but only for the stimulated hand. Indeed, the stimulation modulated size estimation for the stimulated hand (*p* < .05), an effect not seen for face size estimation or non‐stimulated hand (*p* > .05). This was further supported by the effect sizes observed in each group, which were clearly larger for the hand stimulation group, confirming the influence of the stimulation. Specifically, increased somatosensory input caused a reduction of the distortion, confirming bottom‐up modulation of size representation. This type of stimulation activates somatosensory areas (Beste & Dinse, 2013; Pleger et al., 2001; Rode et al., 2012), which appear involved in representing the size of hands. Not only results were statistically significant, but also this was seen as a large effect size. Hence, these results strengthen the link between size representation and somatosensation.

Increased somatosensory information in this case was instrumental for the perceived size of hands, but not for the face. These results confirmed that access to somatosensory and proprioceptive information for hands is prioritised, whereas this may not be the case for the face. For instance, in perceptual competition tasks, visual awareness of the hands is faster when the participant's hand is held in a congruent position to the presented hand images (Salomon et al., 2013). On the contrary, this effect is not seen for faces, in which proprioceptive information does not appear to be as relevant (Korb et al., 2017). These findings support the idea that the face is more visually constructed (Keyes, 2012), and possibly more resilient to plastic changes due to sensory stimulation. Owing to this visual specialisation, the face may need a different timescale for the integration of proprioceptive signals with visual ones, in comparison to hands (Korb et al., 2017). Instead, multisensory information is normally integrated quickly for the hands, with strong visuo‐motor‐proprioceptive relationships (Korb et al., 2017). For example, recent studies have reported the recruitment of the somatosensory cortex in working memory tasks with hand images, in contrast to images of objects, which only recruited visual areas (Galvez‐Pol et al., 2018). Similarly, differences in the integration of multisensory information are also seen between upper and lower limbs, being faster for the hands (van Elk et al., 2013). These results indicate that different sensory modalities will have different relevance depending on the body considered part (Stone et al., 2018).

Competition between sensory modalities helps to understand how different types of information may prime for different tasks. When performing visually guided actions, in which there is visuo‐proprioceptive conflict, the reduction of the involvement of one sensory modality that is not as relevant will, in turn, improve performance. For example, reduction of proprioceptive information through repetitive transcranial magnetic stimulation (rTMS)‐induced deafferentation of the hand can improve mirror drawing (Balslev et al., 2004). In other words, by removing conflicting proprioceptive information, enhanced performance is achieved in the task. Following this, the opposite might be true for the present task. That is, it is possible to hypothesize that increasing somatosensory information in a body part that is constructed more visually will, instead, create more conflict. Furthermore, those who generally rely more on or are more skilled in visual imagery may worsen their performance when the system is ‘overloaded’ with information from a different sensory modality.

Another possible explanation for the lack of effect for face stimulation may be related to the intensity of the stimulation, which was significantly higher for the hands (average of 33.02 mA) compared with the face (average of 17.49 mA). Hence, the lack of significant effect in body size representation after face stimulation may also be stimulation intensity, as in studies testing the forearm (Muret & Dinse, 2018). While it is possible that matching stimulation intensity across groups could provide valuable insights, it is important to note that our primary aim was to assess the impact of stimulation on body representation within the constraints of participant's comfort and tolerance levels. Setting the intensity at the individual maximum tolerable level ensured that stimulation was effective, even if not matched across all groups. In light of these considerations, we believe that the discrepancy in stimulation intensity between groups does not undermine the validity of our findings. Rather, it underscores the practical challenges inherent in conducting sensory stimulation studies across different body parts. Similarly, the limited number of motors for face stimulation may underlie the lack of modulation. However, this variation was necessitated by the need to cover relevant landmarks while considering participant's comfort. The discrepancy of stimulation sites between face and hand underscores the challenges in conducting multisite sensory stimulation research across different anatomical regions, yet it was essential for effective stimulation.

The passive stimulation in this case did not improve motor performance, as seen in the lack of significant results in the peg test for dexterity. Previous studies had found improved motor function after somatosensory stimulation (Kalisch et al., 2008; Wu et al., 2006) due to the associated increase in motor cortical excitability (Kaelin‐Lang et al., 2002; Ridding et al., 2000). Perhaps, a more challenging version of the peg‐test should be implemented that is sensitive enough to the stimulation. Indeed, previous studies identified differences in performance only in the most complex version of the task (Ebied et al., 2004; Kalisch et al., 2008).

As potential limitations of our study, we acknowledge that the method of limits is constrained by its reliance on a binary ‘yes/no’ judgement, which may not fully encompass the complexity of perceived body size while reflecting decisional uncertainty influenced by participants' individual tendencies. However, alternative methods, such as forced‐choice judgements, also have their own set of weaknesses, including potential issues with the comparison of body size between images and uncertainty about whether this accurately reflects true body size (Cornelissen et al., 2019). Similarly, procedures like constant stimuli may require many trials. For all these reasons, the method of limits provided a reasonable compromise between practicality and reliability for our study's objectives.

We further acknowledge that since an *a priori* power calculation was carried out only for the main hypothesis, the effects of secondary analyses were not factored in and the final results may suffer from an under‐power effect where smaller effects may have passed unnoticed. Indeed, the sample size of this study would only allow sufficient power to identify a relatively large effect size (*f* = .8). Hence, our study lacks the power to identify smaller effect sizes.

To sum up, passive sensory stimulation administered through this experimental device has proven its capacity to induce subjective changes of body size without training. It is an inexpensive device, portable, easy to use, that allows different intensities of vibration. This stimulation may have produced associated cortical activation in somatosensory areas, improving the perceived size of the hand. This paradigm may represent an alternative to modulating distorted size representation in patients with body representational deficits.

## AUTHOR CONTRIBUTIONS

**Laura Mora:** Conceptualization; investigation; writing – original draft; methodology; visualization; writing – review and editing; formal analysis; project administration. **Giorgia Committeri:** Conceptualization; writing – review and editing; validation; supervision. **Teresa L'Abbate:** Investigation; writing – review and editing. **Gianna Cocchini:** Conceptualization; methodology; writing – review and editing; formal analysis; supervision; investigation.

## CONFLICT OF INTEREST STATEMENT

The authors declare that there are no conflicts of interest regarding the publication of this manuscript.

## Data Availability

The data that support the findings of this study are available from the corresponding author upon reasonable request.
